# Neuronal Responses to Short Wavelength Light Deficiency in the Rat Subcortical Visual System

**DOI:** 10.3389/fnins.2020.615181

**Published:** 2021-01-06

**Authors:** Patrycja Orlowska-Feuer, Magdalena Kinga Smyk, Anna Alwani, Marian Henryk Lewandowski

**Affiliations:** ^1^Malopolska Centre of Biotechnology (MCB), Jagiellonian University in Kraków, Kraków, Poland; ^2^Department of Neurophysiology and Chronobiology, Jagiellonian University in Kraków, Kraków, Poland

**Keywords:** UV light, blue light, S-cones, melanopsin, rat, subcortical visual system, electrophysiology

## Abstract

The amount and spectral composition of light changes considerably during the day, with dawn and dusk being the most crucial moments when light is within the mesopic range and short wavelength enriched. It was recently shown that animals use both cues to adjust their internal circadian clock, thereby their behavior and physiology, with the solar cycle. The role of blue light in circadian processes and neuronal responses is well established, however, an unanswered question remains: how do changes in the spectral composition of light (short wavelengths blocking) influence neuronal activity? In this study we addressed this question by performing electrophysiological recordings in image (dorsal lateral geniculate nucleus; dLGN) and non-image (the olivary pretectal nucleus; OPN, the suprachiasmatic nucleus; SCN) visual structures to determine neuronal responses to spectrally varied light stimuli. We found that removing short-wavelength from the polychromatic light (cut off at 525 nm) attenuates the most transient ON and sustained cells in the dLGN and OPN, respectively. Moreover, we compared the ability of different types of sustained OPN neurons (either changing or not their response profile to filtered polychromatic light) to irradiance coding, and show that both groups achieve it with equal efficacy. On the other hand, even very dim monochromatic UV light (360 nm; log 9.95 photons/cm^2^/s) evokes neuronal responses in the dLGN and SCN. To our knowledge, this is the first electrophysiological experiment supporting previous behavioral findings showing visual and circadian functions disruptions under short wavelength blocking environment. The current results confirm that neuronal activity in response to polychromatic light in retinorecipient structures is affected by removing short wavelengths, however, with type and structure – specific action. Moreover, they show that rats are sensitive to even very dim UV light.

## Introduction

Every day, the earth spins around its axis producing considerable changes in the amount and spectral composition of environmental light. Whilst changes in irradiance are easily noticed by humans, spectral differences are less obvious. The anthropocentric definition of visible light describes it as an electromagnetic radiation between 380 and 760 nm, however, caution must be taken when considering other organisms possessing different photoreceptors, thus “seeing” light in different ranges (Sliney, 2016). A typical daylight spectrum is almost indistinguishable from the full moon spectrum, whereas irradiance varies between them by several magnitudes (Johnsen et al., 2006; Walmsley et al., 2015). On the other hand, sunset and twilight are the most crucial moments of the day when spectral irradiance (significantly higher irradiance of particular wavelengths) substantially changes. When sun reaches the horizon (solar elevation decreases from 10 to 0°) the spectral composition of light changes from being long-wavelength shifted to more neutral and further (from 0 to −10°) to short-wavelengths enriched (Johnsen et al., 2006; Walmsley et al., 2015).

Excellent examples of ‘seeing light differently’ animals are nocturnal rodents (e.g., rats and mice). In contrast to primates (who are tetrachromats), these rodents are dichromatic and possess three classes of retinal photoreceptors: rods, cones (S-cones and M-cones) and intrinsically photosensitive retinal ganglion cells (ipRGCs). These photoreceptors are maximally sensitive to 498 (rod opsin), 359 (ultraviolet opsin, UVS, S-cones), 509 (middle wavelength opsin, MVS, M-cones) and 480 (melanopsin) nm light, respectively, (Jacobs et al., 2001; Berson et al., 2002). Due to the expression of UVS opsin and lack of long wavelength opsin (LVS), rodents are more sensitive to UV light and less to longer wavelengths compared to primates (Jacobs et al., 2001).

Several different brain structures, such as the dorsal and ventral lateral geniculate nuclei (dLGN, vLGN), suprachiasmatic nucleus (SCN), olivary pretectal nucleus (OPN) and superior colliculus (SC), receive light information from the retina and thus control image and non-image forming (NIF) visual functions. Experiments on genetically modified animals have shown that all photoreceptor types are required for proper functioning of the visual system and biological clock. However, a simplified view is that rods and cones are more important for vision, whereas melanopsin cells for NIF functions, such as circadian entrainment, pupillary light reflex (PLR) and hormone secretion (Lucas and Foster, 1999; Berson et al., 2002; Gooley et al., 2003; Güler et al., 2008; Ecker et al., 2010; Brown et al., 2010; Allen et al., 2011, 2017; Brown et al., 2012; for review see Duda et al., 2020). Photoreceptor contribution to these functions has been studied in more detail using the method of silent substitution whereby precisely controlling the output of several primaries allows for the independent control of photoreceptor excitation (Allen and Lucas, 2016; Allen et al., 2017; Hayter and Brown, 2018; Spitschan and Woelders, 2018; Woelders et al., 2018). Although, these studies led to crucial findings, they are limited in that they can only be conducted in restricted laboratory-based conditions, thus making it highly challenging (at least for now) to use this methodology in a natural/real world scenario (Duda et al., 2020). An alternative approach to study light influence on NIF functions in behavioral studies is with the use of filtered light. Due to melanopsin’s substantial contribution to all NIF functions the most widely used filters are cut off/bandwidth filters and amber lenses/goggles that remove short wavelengths from polychromatic/visible light (Rahman et al., 2008, 2017; Wren et al., 2014; Gladanac et al., 2019). Interestingly, these approaches of filtering have also been successfully exploited in minimizing disruptive effects of night time light exposure on circadian rhythms, quality of sleep and cognitive performance in humans (Gringras et al., 2015; Ayaki et al., 2016; Ostrin et al., 2017; Mortazavi et al., 2018; Kazemi et al., 2019; Domagalik et al., 2020). However, until now an unanswered question remains: how do similar changes in the spectral composition of light influence neuronal activity in retinorecipient brain structures?

Therefore, we decided to examine how light-induced neuronal activity in the rat subcortical visual system is affected by changing spectral composition of light (removing 90% of short-wavelength from polychromatic white light). We have investigated three structures involved in different physiological functions: the dLGN (engaged in classic vision formation), OPN (responsible for PLR) and SCN (the central pacemaker of the circadian timing system).

## Materials and Methods

### Ethical Approval

Experimental procedures were approved by the ethics committee of Jagiellonian University in Krakow (permission no.: 25/2013) and complied with the Polish Animal Protection Law. All experiments were conducted in accordance with regulations and standards of the Directive 2010/63/EU of the European Parliament, and of the Council of 22 September 2010 on the protection of animals used for scientific purposes, the 3Rs law, and the ARRIVE guidelines for reporting experiments involving living animals with respect to anesthesia and animal handling (Kilkenny et al., 2010).

### Animals

The study was performed on 13 adult male Long Evans rats (2–4 months old, weighing 270 – 400 g) bred in the animal facility of the Institute of Zoology and Biomedical Research at Jagiellonian University in Krakow. Animals were group-housed under 12:12-h light:dark cycle at a temperature of 22 – 23°C and ∼60% humidity with free access to food and water.

### Anesthesia and Surgery

General anesthesia was provided by an intraperitoneal injection of urethane (1.5 g/kg dissolved in 2 mL saline; Sigma-Aldrich, Germany). The level of anesthesia was verified by the lack of withdrawal and ocular reflexes and an additional dose of urethane (10 – 20% of initial dose) was supplied when required. The body temperature was monitored and automatically maintained at 37 ± 0.5°C by a homeothermic control. Experiments were conducted during two different light regimes: light phase (ZT 1-10; dLGN and OPN recordings) and dark phase (ZT 13-22; SCN recordings). SCN recordings were conducted during the dark phase due to maximum light responsiveness of its neurons at that phase (Meijer et al., 1998; Brown et al., 2011).

Animals’ heads were secured in a stereotaxic frame via ear and incisor bars (Advanced Stereotaxic Instruments, United States). The skull surface was exposed and stereotactic points: *bregma* and *lambda* were set. The coordinates were determined according to the rat stereotaxic brain atlas (Paxinos and Watson, 2007). A craniotomy was drilled above the dLGN (3.7 mm lateral, 4.6 mm posterior from *bregma*), OPN (1.2 mm lateral, 4.8 mm posterior from *bregma*) or SCN (1.0 mm lateral, 0.5 mm posterior from *bregma*). After removing the dura, the brain surface was covered with mineral oil (Sigma-Aldrich, Germany).

Silicon arrays from Neuronexus Technologies Inc., (United States) consisting of 32 channels (4 shanks spaced 200 μm) were used for all *in vivo* recordings (dLGN and OPN: A4 × 8–10 mm-50-200-177-A32; SCN: Buzsaki 32L). Probes were dipped in a fluorescent dye (CM-DiI; Invitrogen, United Kingdom), centred at *bregma* point, moved above the craniotomy according to the coordinates and lowered into the brain (to the depth of 5.0, 4.8, or 10.00 mm from the brain surface in the case of the dLGN, OPN, and SCN, respectively) by using a one-axis oil hydraulic micromanipulator (model: MO-10; Narishige Inter- national Ltd., Japan). For the SCN recordings probes were mounted at a 5° angle.

Signals (ampilified ×3,000) were acquired using the OmniPlex D Neural Data Acquisition System (Plexon, Inc., United States), high-pass filtered (0.05 Hz), digitized at a rate of 40 kHz and then stored for offline analysis. Once the recording probe was in place, the Faraday cage was covered with a light impermeable material (Ex-lite fabric; Domarant, Poland) and rats were left for ∼30 min to dark adapt and stabilize neuronal activity.

### Light Stimulation

**FIGURE 1:**
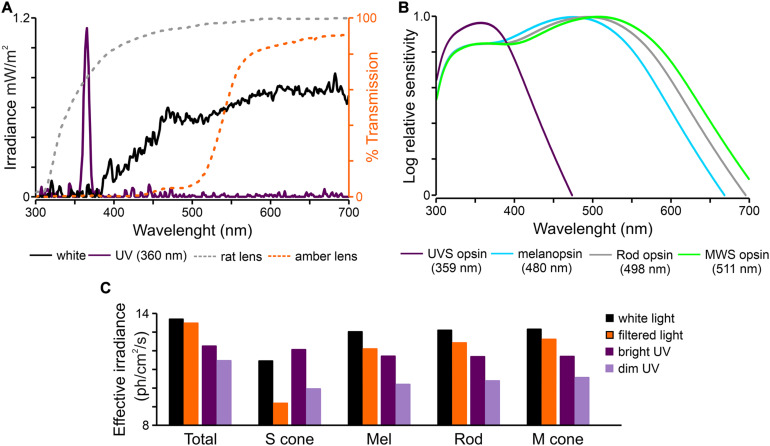
Characteristic of light stimuli used in the study. **(A)** Spectral transmission profiles of polychromatic white and monochromatic UV (360 nm) light used in the present study and corresponding transmission of rat lens (Jacobs et al., 2003) and amber lens (Prima 67 or Igel RX SPHERE; UltraVision, United Kingdom). **(B)** Overlapping spectral sensitivity of rat photoreceptors. **(C)** Effective irradiances for each type of photoreceptors produce by light stimuli presented in panel **(A)** (Lucas et al., 2014). UV – monochromatic light (360 nm).

The eye contralateral to the craniotomy was held open and the pupil was dilated with topical application of atropine solution (Atropinum Sulfuricum WZF 1%, Polfa, Poland). An amber contact lens (commercially available Prima 67 or Igel RX SPHERE; UltraVision, United Kingdom) was used to reduce the transmittance of short wavelengths light by ∼90% (Figure 1A). It was carefully put on the rat’s eye ensuring its full coverage and moisturized with mineral oil (Sigma-Aldrich, Germany). Full-field light stimulations were delivered by a custom-made light source consisting of individually controlled monochromator (320 – 1,000 nm) and 150 W Xenon lamp (Instytut Fotonowy, Poland). The light source was positioned ∼1 cm from the eye. White light and UV (360 nm, Figure 1A) pulses (6–10×: 15 s-long, 45 s interstimulus interval) were presented to the eye before and after applying the amber contact lens (Figure 1A). Light was measured using a calibrated spectroradiometer (BLACK-Comet-SR spectrometer; StellarNet Inc., United States). Spectral power densities were converted to effective irradiance for each of the rat photoreceptors (Lucas et al., 2014) by multiplying by the normalized *in vivo* spectral sensitivity for each photopigment and correcting for the pre-receptoral filtering (Jacobs et al., 2003) across the spectrum (Figures 1A–C). The irradiance of filtered light was calculated by applying a transmittance curve for the amber lens (Figure 1A) on top of rat lens.

### Histological Verification

After recording, rats were euthanized by overdose of sodium pentobarbital (Biovet, Poland). Brains were removed from the skull and transferred to PFA for ∼48 h. Coronal slices (100 μm thick) were cut using a vibroslicer (Leica VT1000S, Germany). CM-DiI marks were verified with a Zeiss fluorescent microscope (Zeiss Axio Imager, M2, United States). Recording sites were estimated based on the atlas of Paxinos and Watson, 2007 by using the optic chiasm, 3rd ventricle and hippocampus as landmarks.

### Data Analysis

Spike sorting was conducted offline using Offline Sorter (Plexon, United States). Waveforms extracted from the continuous signal (typically 40 μV) were sorted using Template Matching Method and Principal Component Analysis (PCA) and then verified manually. Reliable single unit isolation was confirmed by referenced to MANOVA F statistics, J3 and Davies-Bouldin validity metrics (Offline sorter) and by monitoring interspike interval histograms (>1 ms). Special care was taken to ensure that no cell was included more than once in the SCN recordings due to the use of Buzsaki electrode (cross correlogram analysis of unit firing was verified).

Cells were classified based on their responses to polychromatic white light presented from darkness (total photons: 4.87 × 10^13^ photons/cm^2^/s corresponding to 51.45 μW/cm^2^). Peristimulus time histograms (PSTHs, bin size = 0.1 s) were calculated using Neuroexplorer (Nex Technologies, United States) to identify the light responsive neurons. Responses were considered significant when the mean firing rate during the onset/offset peak activity (0–0.5 s) and tonic component of the response (the last 5 s of the stimuli) exceeded the pre-stimulus firing activity (3 s epoch before the stimulus onset) by 2× standard deviations (SD). Next, cells were classified as transient ON, transient OFF, sustained and suppressed depending whether their firing rate during different phases of the response followed the 2× SD rule.

Different stimuli evoked different response types, thus cells were also categorized based on that feature. Three cell types were distinguished here: stable, changing and non-responsive. Stable cells kept the same type of responses upon different stimuli (e.g., white vs filtered light; either being always transient ON, transient OFF, sustained, or suppressed), changing cells adapted their response profile (changing between two response types), whereas non-responsive stopped responding after switching from white/UV to filtered light stimuli.

All statistical analyses were performed using GraphPad Prism 5 (GraphPad Software, Inc., United States). Two-way rmANOVA followed by a Bonferroni *post hoc* test, Student’s paired *t*-test and paired Wilcoxon test (for non-parametrical data) were used to estimate differences between datasets. The irradiance coding properties were assessed by fitting the data to the three-parameter sigmoidal curve according to the Hill law. The results are reported as mean ± SEM.

## Results

The main aim of this work was to determine how changes in the light spectrum influence light-induced activity in image forming (dLGN) and NIF (OPN and SCN) brain centers. We focused on the short wavelengths light by using commercially available amber contact lenses reducing transmittance of UV/blue light by 90% (Figure 1).

### Classification of Light Response Types

**FIGURE 2:**
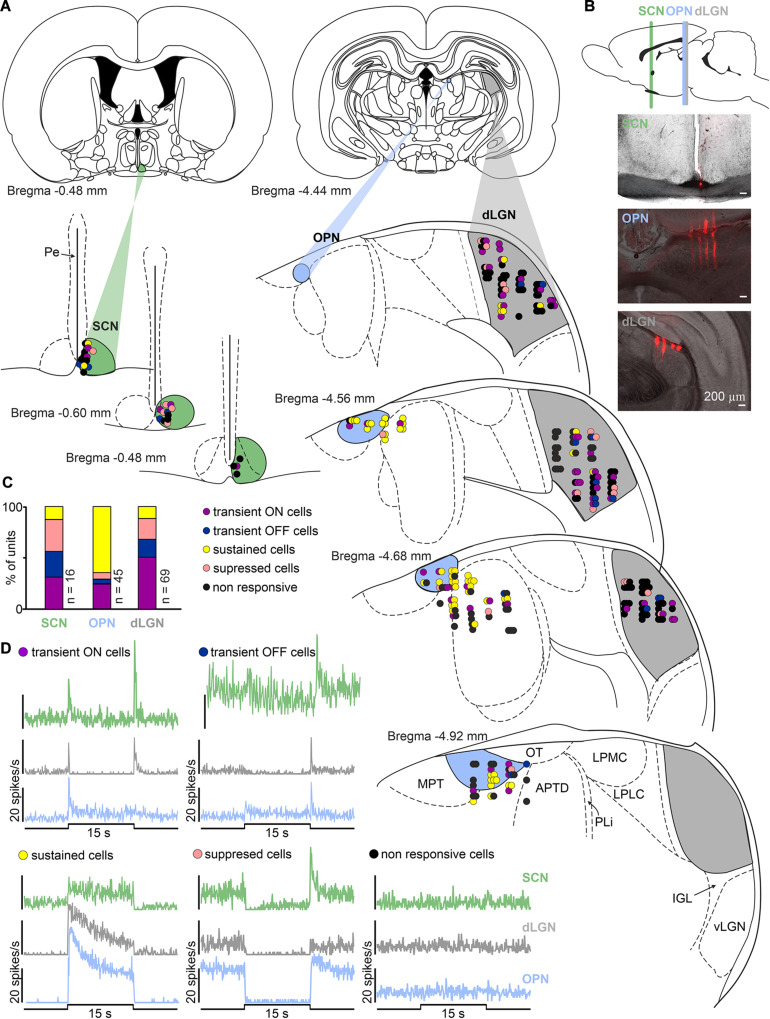
Diversity of light responsive cells in the black hooded rat subcortical visual system. **(A)** Schematic coronal slices at various distances from *bregma* with anatomical localization of all recorded cells in the SCN, OPN, and dLGN (color coded) according to the rat brain stereotaxic atlas (Paxinos and Watson, 2007). **(B)** Exemplary images showing multielectrode placements in the region of SCN, OPN and dLGN. **(C)** Proportion of different types of light responsive neurons recorded in each structure investigated. **(D)** Four types of light responsive cells detected in the SCN, OPN and dLGN. Traces represent responses to 15 s polychromatic white light steps from dark (bin size = 1 s; total photons: 4.87 × 10^13^ photons/cm^2^/s corresponding to 51.45 μW/cm^2^). SCN, suprachiasmatic nucleus; OPN, olivary pretectal nucleus; dLGN, dorsal lateral geniculate nucleus; vLGN, ventral lateral geniculate nucleus; IGL, intergeniculate leaflet; OT, nucleus of the optic tract; MPT, medial pretectal nucleus; APTD, anterior pretectal nucleus (dorsal part); PLi, posterior limitans thalamic nucleus; LPMC, lateral posterior thalamic nucleus (mediocaudal part); LPLC, lateral posterior thalamic nucleus (laterocaudal part).

In multi-unit recordings, 158 light responsive cells were identified: 69 out of 181, 73 out of 108 and 16 out of 28, in the dLGN, pretectum (OPN: 45 out of 64) and SCN, respectively. Figure 2 shows schematic localization of all recorded cells based on histology and verification with the rat brain atlas (Paxinos and Watson, 2007). Light responsive cells showed reproducible responses to a bright polychromatic white light stimulus (total photons: 4.87 × 10^13^ photons/cm^2^/s corresponding to 51.45 μW/cm^2^) and their firing rate exceeded 2 × SD at the light onset/offset (peak activity: 0 – 0.5 s) and/or during the last 5 s of the stimulus (tonic component). Next, cells were categorized as sustained, transient ON, transient OFF and suppressed (Figures 2C,D) based on the mean firing rate during different phases of the response. High firing rate during the last 5 s of the stimulus (≥2 × SD) classified cells as sustained. Transient ON cells exhibited short peaks of activity at light onset and occasionally offset, however, the firing rate during light presentation was at the level of baseline spiking. Transient OFF cells were characterized by peak activity exclusively at light offset. The fourth class: suppressed cells, exhibited a light inhibition of firing rate during pulse presentation (below baseline). All four response profiles were found in each of the investigated areas. The most prevalent profiles in each area were: dLGN, transient ON cells; OPN, sustained; SCN, transient OFF and suppressed (Figure 2C).

### Removing Short Wavelengths From White Light Attenuates Light-Induced Activity in the Image Forming Brain Structure – dLGN

We first aimed to compare responses to white and filtered light stimulation. To achieve this goal amber lenses removing 90% of short wavelengths (Figure 1) were used. Amber lenses reduced the effective irradiance for S-cones by two orders of magnitude (from 11.44 to 9.17 log photons/cm^2^/s), and for melanopsin by one order of magnitude (from 13.02 to 12.09 log photons/cm^2^/s; Figure 1C). A change in firing rate before (3 s baseline), during and after light stimulation was calculated and three different phases of the response were compared: onset and offset peak activities (0 – 0.5 s) and tonic component (10 – 15 s). By comparing these three phases in different cell types, we aimed to reveal the extent to which short wavelength light influences light-induced activity derived from different photoreceptors (namely amplitude of peak activity for cones/rods and amplitude of tonic component for melanopsin).

**FIGURE 3:**
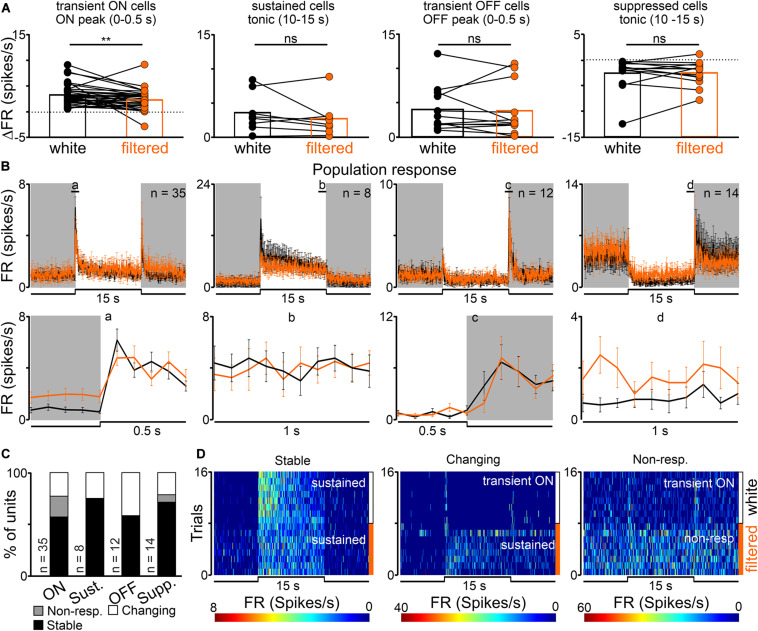
Effects of removing short wavelength from polychromatic white light on neuronal activity in the rat dLGN. **(A)** A change in firing rate (peak activity for transient cells and tonic component for sustained cells) in response to polychromatic white light and filtered polychromatic light for different types of light responsive cells in the rat dLGN (indicated above the histograms). **(B)** Peri stimulus time histograms (PSTH): upper traces represent mean ± SEM (bin size = 0.1 s) responses to 15 s long, white and filtered stimuli (total photons: white: 4.87 × 10^13^ photons/cm^2^/s; filtered: 3.06 × 10^13^ photons/cm^2^/s) of all units corresponding to each class; bottom traces represent higher resolution of 1 s long trace marked with rectangles above the PSTHs (the peak onset and offset for transient cells and tonic component for sustained and suppressed cells, respectively). **(C)** Histograms showing the proportion of stable, changing their response profile and not responding to filtered light cells across the four different types of light responsive cells recorded in the dLGN. Examples of such cells are shown as heat maps in **(D)**. Data in **(A)** were analyzed by Wilcoxon matched-pairs signed rank test. **p < 0.01; ns, non-significant.

A statistically significant decrease in the amplitude of peak, but not tonic part of the response, (from 3.26 ± 0.35 to 2.27 ± 0.33 Hz) was observed in transient ON cells in the response to white vs filtered light stimuli (peak activity: Wilcoxon matched-pairs signed rank test, two-tailed, *p* = 0.0021, *W* = −366.0; tonic component: Wilcoxon matched-pairs signed rank test, two-tailed, *p* = 0.4697, *W* = 86.0, *n* = 35; Figures 3A,B). A similar tendency was observed for sustained cells (peak activity: Wilcoxon matched-pairs signed rank test, two-tailed, *p* = 0.0781, *W* = −26.0, *n* = 8; tonic component: Wilcoxon matched-pairs signed rank test, two-tailed, *p* = 0.3828, *W* = −14.0, *n* = 8; Figures 3A,B). The firing rate of transient OFF and suppressed cells was not influenced by change in the light spectrum (Figures 3A,B). These observed changes only in peak amplitude suggest that transient ON and sustained cells in the dLGN received information from S-cones, as irradiance was reduced by two logs in the above protocol for S-cones (Figure 1C).

Next, response profiles to white and filtered light stimulations were compared to verify whether filtering short wavelengths from the light spectrum alters this feature. The majority of cells (43/69, 62%) exhibited the same response profile to both stimulations, 12% of cells (8/69) stopped responding and 26% had an altered (18/69) response profile (Figures 3C,D). These data further suggest that cells not responding to filtered light potentially receive light information exclusively from S-cones, whereas cells adapting their response profile might reflect irradiance-dependent responses connected with blocking short wavelengths (Tikidji-Hamburyan et al., 2014).

### UV Sensitivity of the Rat dLGN

Additionally, we sought to establish whether the rat dLGN is responsive to monochromatic UV light stimulation (360 nm), and by using the amber lenses, verify how sensitive it is to short wavelengths (S-cones effective irradiance was 12.07 and 9.95 log photons/cm^2^/s, for UV and filtered UV stimulation, respectively).

In total, 63 cells were responsive to UV light and all four response profiles were observed: sustained (*n* = 6), transient ON (*n* = 27), transient OFF (*n* = 13) and suppressed (*n* = 17). Interestingly, some of white light responding cells did not respond to UV stimulation (*n* = 8), and three of tested cells responded only to UV light, implying that cells in the dLGN receive input from different photoreceptors amongst which are S-cones. It is important to note that the polychromatic white light source used in the present study emits minimally in the UV range (Figure 1A), therefore it can be predicted to minimally excites rat S-cones. It is therefore possible that not all neurons receiving retinal input from S-cones were detected in this protocol.

**FIGURE 4:**
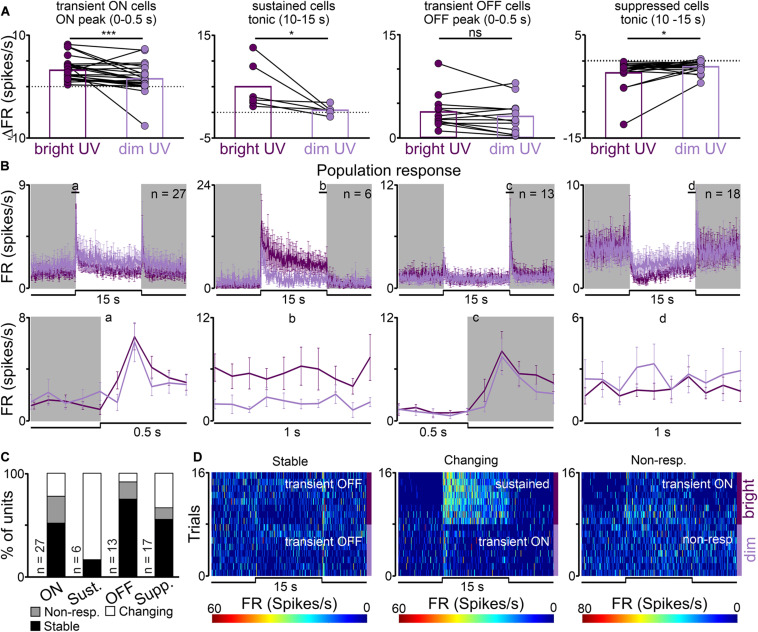
UV sensitivity of the rat dLGN. **(A)** A change in firing rate (peak activity for transient cells and tonic component for sustained cells) in response to monochromatic bright and dim UV light (UV) for different types of light responsive cells in the rat dLGN (indicated above histograms). **(B)** Peri stimulus time histograms (PSTH): upper traces represent mean ± SEM (bin size = 0.1 s) responses to 15 s long, bright and dim UV stimuli (360 nm; S-cones photons: bright: 1.16 × 10^12^ photons/cm^2^/s, dim: 8.95 × 10^9^ photons/cm^2^/s) of all units corresponding to each class; bottom traces represent higher resolution of 1 s long trace marked with rectangles above the PSTHs (the peak onset and offset for transient cells and tonic component for sustained and suppressed cells, respectively). **(C)** Histograms showing the proportion of stable, changing their response profile and not responding to dim UV stimuli cells across the four different types of UV responsive cells recorded in the dLGN. Examples of such cells are shown as heat maps in **(D)**. Data in **(A)** were analyzed by Wilcoxon matched-pairs signed rank test. *p < 0.05; ***p < 0.001, ns, non-significant.

In the response to bright vs dim UV light statistically significant decreases were observed in the amplitude of peak activity of transient ON cells (from 3.19 ± 0.43 to 1.52 ± 0.53 Hz, Wilcoxon matched-pairs signed rank test, two-tailed, *p* = 0.0002, *W* = −293.0, *n* = 27, Figures 4A,B), tonic component of sustained cells (from 5.01 ± 1.90 to 0.42 ± 0.37 Hz, Wilcoxon matched-pairs signed rank test, two-tailed, *p* = 0.0313, *W* = −21.0, *n* = 6, Figures 4A,B) and increase in the tonic component of suppressed cells (from −2.29 ± 0.68 to −1.11 ± 0.27 Hz, Wilcoxon matched-pairs signed rank test, two-tailed, *p* = 0.0472, *W* = 91.0, *n* = 18, Figures 4A,B). There were no significant changes in the amplitude of offset peak activity of transient OFF cells (Wilcoxon matched-pairs signed rank test, two-tailed, *p* = 0.1465, *W* = −43.0, *n* = 13, Figures 4A,B).

Our next aim was to compare light response profiles between bright and dim UV light stimuli. Similarly, like in the white/filtered white stimuli, three main classes of cells were identified: responding in the same way to both stimuli (34/63, 54%), not responding to filtered light (11/63, 17%) and changing their response profile (18/63, 29%). Examples of each class are presented in Figures 4C,D. Interestingly, the majority of cells responded to dim UV light which irradiance was at the level of 9.95 log photons/cm^2^/s, implying that the rat dLGN is very sensitive to UV light.

### Does Changing the Light Spectrum Influence Irradiance Coding Properties of OPN Neurons?

Next, we decided to perform a similar experiment in a NIF structure – the OPN. The OPN is well-known for its role in the PLR (Trejo and Cicerone, 1984) and its ability to track changes in irradiance (Allen et al., 2011; Szkudlarek et al., 2012). Both these functions have been shown to rely on melanopsin and these cells provide a major retinal input to the OPN. Thus, a question arises whether removing short wavelengths from the white spectrum influences the ability of OPN neurons to encode light intensity. Therefore, we extended our protocol and used increasing irradiance steps presented with and without the amber lens to address this question.

**FIGURE 5:**
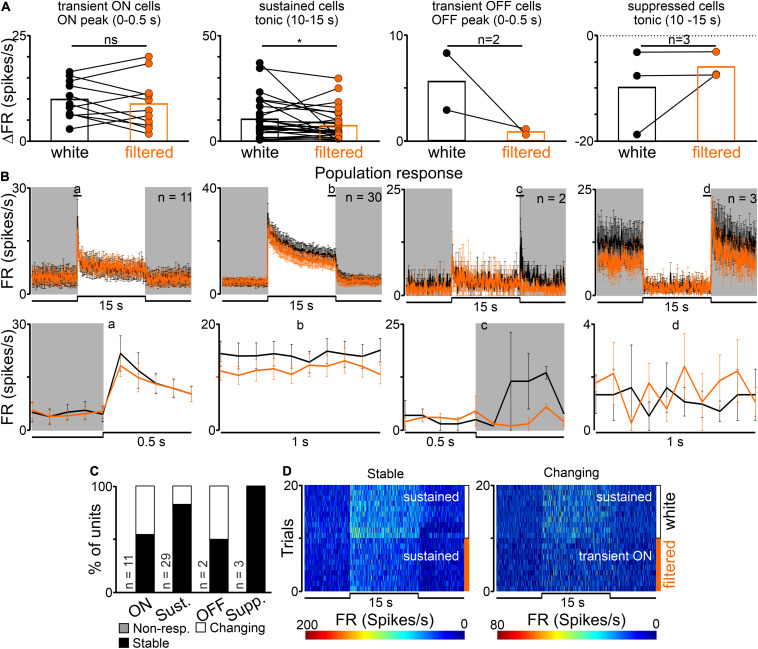
Effects of removing short wavelength from polychromatic white light on neuronal activity in the rat OPN. **(A)** A change in firing rate (peak activity for transient cells and tonic component for sustained cells) in response to polychromatic white light and filtered polychromatic light for different types of light responsive cells in the rat OPN (indicated above histograms). **(B)** Peri stimulus time histograms (PSTH): upper traces represent mean ± SEM (bin size = 0.1 s) responses to 15 s long, white and filtered stimuli (total photons: white: 4.87 × 10^13^ photons/cm^2^/s; filtered light: 3.06 × 10^13^ photons/cm^2^/s) of all units corresponding to each class; bottom traces represent higher resolution of 1 s long trace marked with rectangles above the PSTHs (the peak onset and offset for transient cells and tonic component for sustained and suppressed cells, respectively). **(C)** Histograms showing the proportion of stable, changing their response profile and not responding to filtered light cells across the four different types of light responsive cells recorded in the OPN. Examples of such cells are shown as heat maps in **(D)**. Data in **(A)** were analyzed by Wilcoxon matched-pairs signed rank test and Paired *t*-test. *p < 0.05; ns, non-significant.

Mostly sustained (*n* = 29/45) and transient ON (*n* = 11/45) cells were recorded in the OPN, whereas suppressed (*n* = 3/45) and transient off (*n* = 2/45) were very rare (Figure 2), in agreement with previous reports (Zhang et al., 1996; Allen et al., 2011; Szkudlarek et al., 2012). In contrast to our observations in the dLGN, there were no significant differences in the amplitude of onset peak activity in transient ON cells, however, decreased activity was observed in the amplitude of both, onset peak and tonic component of sustained cells between white and filtered stimuli (transient ON: Paired *t*-test, two-tailed, *p* = 0.4194, *t* = 0.8422, df = 10, *n* = 11; sustained peak: Paired *t*-test, two-tailed, *p* = 0.0318, *t* = 2.256, df = 29, *n* = 30; sustained tonic: Wilcoxon matched-pairs signed rank test, two-tailed, *p* = 0.0145, *W* = −235.00, *n* = 30; Figures 5A,B). These data suggest that short wavelength might be particularly important for NIF functions associated with sustained responses in the OPN.

Moreover, the majority of cells (34/45, 76%) responded similarly to both white and filtered stimuli, however, changes in the response profile (11/45, 24%) were also observed. Interestingly, changes occurred in both sustained and transient ON groups and cells transformed into transient ON and sustained, respectively, (Figures 5C,D). However, it has to be emphasized that changes in the direction from transient ON to sustained response profile were very subtle (at the level of the set threshold – 2 × SD rule). Importantly, there were no cells which stopped responding to filtered light to some degree suggesting that S-cones input to the OPN may be weaker than to the dLGN.

**FIGURE 6:**
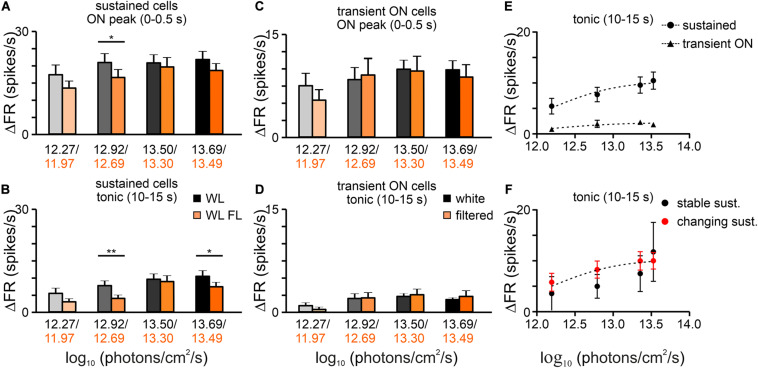
Irradiance coding properties of OPN cells. Bar graphs show the mean changes in the peak activity (±SEM) and tonic component (±SEM) of sustained **(A,B)** and transient ON **(C,D)** cells, calculated as a difference between adequate phase of the response and pre-stimulus activity (3 s). The mean activity was compared both in terms of the light spectrum (white and filtered light) and increasing intensity of the stimulus (irradiance). **(E)** Log:linear curves (three parameters) were fitted to the tonic component of the responses of sustained and transient ON cells. According to the results of the *F*-test, the data were best fitted with separate functions (*F*-test, *P* < 0.0001). **(F)** Log:linear curves (three parameters) were also fitted to the tonic component of the responses of stable and changing their response profile in response to filtered light sustained cells. *F*-test show that the data are best fitted with the same function (*F*-test, *p* = 0.5540). Data in **(A–D)** were analyzed by two-way rmANOVA test followed by Bonferroni’s multiple comparison test. *p < 0.05, **p < 0.01

Next, four irradiance steps were compared between white and filtered light. The white light effective irradiance for total photons were in the range 12.27 to 13.69 log photons/cm^2^/s, thus above the melanopsin activation threshold (Panda et al., 2003; Wong, 2012). Significant differences in the light induced activity were observed in both irradiance steps and spectral composition of light in the amplitude of peak as well as in tonic component of sustained cell responses (peak: two-way rmANOVA, I factor: irradiance: *p* < 0.0001, II factor: light spectrum: *P* = 0.0164; Interaction irradiance x spectrum: *p* = 0.5565; *n* = 30, Bonferroni’s multiple comparison test; tonic: two-way rmANOVA, I factor: irradiance: *P* < 0.0001, II factor: light spectrum: *P* = 0.0158; Interaction irradiance × spectrum: *p* = 0.2907; *n* = 30, Bonferroni’s multiple comparison test, Figures 6A,B). The same analysis was performed for transient ON cells and only changes in the irradiance were found to be significant (Figures 6C,D).

We were not able to compare the response curves for the two conditions, as the amber lens not only changed the spectrum of light but also effective irradiance (Figure 1). Thus, to determine whether sustained cells that had stable and changing response profiles to white and filtered light (assessed for the highest light irradiance used) exhibit a difference in ability to code white light irradiance, log response curves (three parameters) were fitted to the data. Surprisingly, both cell types could be fitted with the same curve (*F*-test; p = 0.5540; Figure 6F). In contrast, sustained cells and transient ON cells are fitted with different curves (*F*-test; *p* < 0.0001; Figure 6E).

**FIGURE 7:**
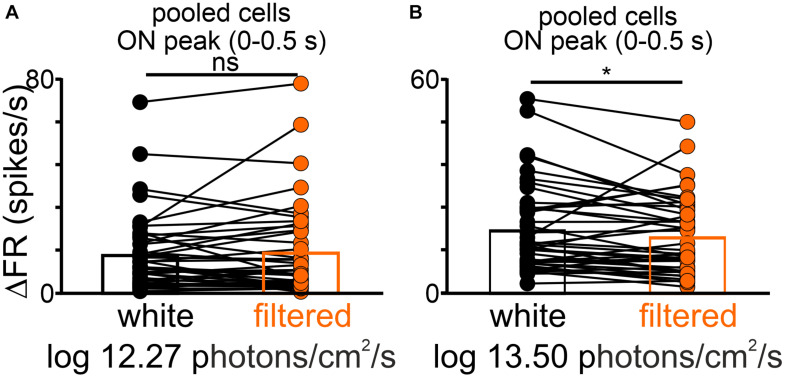
The difference in the peak activity of light responsive cells in the OPN under different spectra. A change in firing rate (peak activity for pooled cells: transient ON and sustained cells) in response to polychromatic white light and filtered polychromatic light with matched intensities **(A)** log 12.27 and **(B)** 13.50 photons/cm^2^/s. Data in **(A,B)** were analyzed by Wilcoxon matched-pairs signed rank test. *p < 0.05, ns, non-significant.

As a final test, changes in the peak responses of the pooled dataset (transient ON and sustained cells) were compared between equal light intensities (total photons; two irradiances: log 12.27 and log 13.50 photons/cm^2^/s) but different spectra (white vs filtered light). Interestingly, a significantly lower firing rate was observed for the filtered light of log 13.50 photons/cm^2^/s, suggesting that some OPN neurons receive S-cone input (Wilcoxon matched-pairs signed rank test, two-tailed, *p* = 0.0294, *W* = −335.0, *n* = 41, Figure 7).

### SCN Is Sensitive to UV Light

After (1) confirming altered light induced activity in transient ON dLGN cells and sustained OPN cells in response to filtered light, and (2) showing that the rat dLGN is sensitive to UV light stimuli we attempted to verify whether similar results can be obtained in the main NIF structure responsible for circadian entertainment – the SCN. Thus, exactly the same protocol as for the dLGN was repeated in the SCN. Recordings in the SCN were conducted during the dark phase due to maximal neuronal responsiveness to light at this phase. It has previously been reported that during the light phase light sensitive neurons in the SCN are difficult to find, have lowered response amplitude and significantly reduced cone-input that was especially crucial to the aim of the current study (Meijer et al., 1989; Brown et al., 2011). In contrast, light evoked firing in the dLGN is not a subject of such changes (Brown et al., 2011) and we are not aware of any data comparing light responses between light and night phase in the OPN.

**FIGURE 8:**
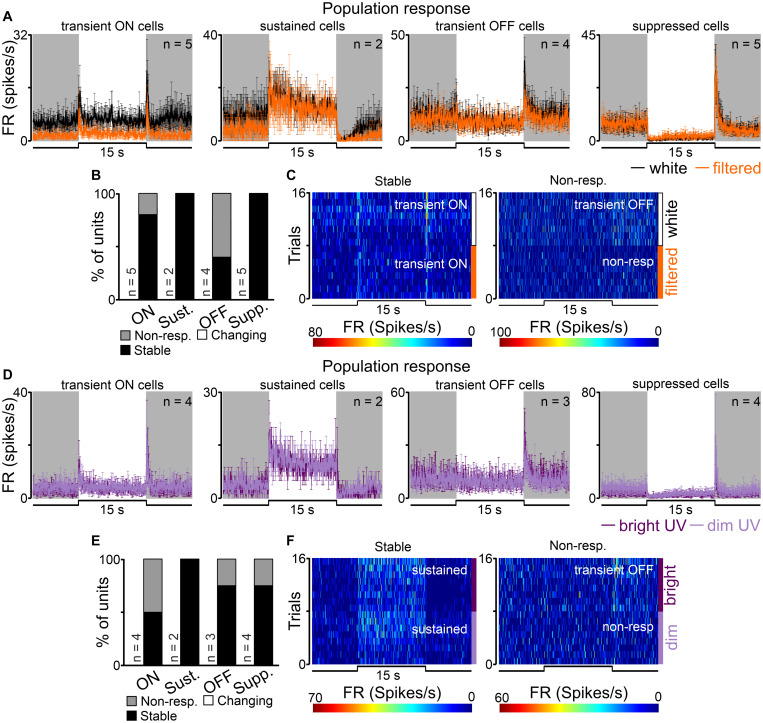
Populational responses to short wavelengths filtered and UV light in the rat SCN. **(A)** Peri stimulus time histograms (PSTH): upper traces represent mean ± SEM (bin size = 0.1 s) responses to 15 s long, white and filtered stimuli (total photons: white: 4.87 × 10^13^ photons/cm^2^/s; filtered: 3.06 × 10^13^ photons/cm^2^/s) of all units corresponding to each class. **(B)** Histograms showing the proportion of stable, changing their response profile and not responding to filtered light cells across the four different types of light responsive cells recorded in the SCN. Examples of such cells are shown as heat maps in **(C)**. **(D)** Peri stimulus time histograms (PSTH): upper traces represent mean ± SEM (bin size = 0.1 s) responses to 15 s long, bright and dim UV stimuli (360 nm; S-cones photons: bright: 1.16 × 10^12^ photons/cm^2^/s, dim: 8.95 × 10^9^ photons/cm^2^/s) of all units corresponding to each class. **(E)** Histograms showing the proportion of stable, changing their response profile and not responding to dim UV stimuli cells across the four different types of UV responsive cells recorded in the dLGN. Examples of such cells are shown as heat maps in **(F)**. Data were not statistically tested due to low *n*.

As expected, by the size of the nuclei and their locations in the brain (Figure 2), the number of units recorded in the SCN was lower than in the dLGN and OPN, however, all four types of cells were recorded. Surprisingly, transient cells were the most frequently observed (9/16), while sustained cells were very rare (*n* = 2) (Figure 2). Even though, transient ON, transient OFF and suppressed cells decreased their activity in response to filtered light, it could not be supported statistically due to the group size (Figure 8A).

The majority (12/16, 75%) of recorded cells did not change their response profile between white and filtered stimuli (Figures 8B,C). However, the remaining cells (4/16, 25%, all in the transient group) did not respond to filtered light suggesting S-cones input to the SCN.

Next, we moved to compare responses to UV (360 nm; log 12.07 and 9.95 photons/cm^2^/s) stimulations in the SCN. Overall, 13 cells were classified as responsive to UV light and four response profiles were found: transient ON (*n* = 4), sustained (*n* = 2), transient OFF (*n* = 3) and suppressed (*n* = 4) as shown in Figure 8D. Similarly like in the dLGN dataset, we found cells which only responded to white light stimuli (*n* = 3) and one cell that only responded to UV light. In response to bright *vs* dim UV stimuli a tendency toward decrease the amplitude of onset peak activity from 8.58 ± 2.06 to 5.69 ± 1.92 Hz was observed in the pooled dataset for transient ON and sustained cells (peak response: Wilcoxon matched-pairs signed rank test, two-tailed, *p* = 0.0625, *W* = −19.0, *n* = 6).

Comparison of response profiles between UV stimuli revealed that the majority of cells responded similarly to both stimuli (9/13, 69%, Figures 8E,F) and a minority of them stopped responding (4/16, 31%, Figures 8E,F). Based on this data, and the observation that some cells responded to quite dim stimuli (9.95 log photons/cm^2^/s for S-cones) it seems reasonable to suggest that the rat SCN is very sensitive to UV light.

## Discussion

The present study reveals how changes in the spectral composition of light influence light-induced neuronal activity in the rat subcortical visual system. Three structures were investigated in detail: the dLGN (involved in vision), the OPN and the SCN (responsible for NIF visual functions) (Moore and Lenn, 1972; Trejo and Cicerone, 1984; Guido, 2018).

### Light Responsive Cells in the Pigmented Rat dLGN, SCN, and OPN

To our knowledge this is the first comprehensive study describing light responsive neurons in the subcortical visual system of the black hooded *Long Evans* rat. Previously published data investigating NIF regions were mostly collected from wild type mice [SCN: (Groos and Mason, 1980; Brown et al., 2011; van Diepen et al., 2013; Walmsley and Brown, 2015); OPN: (Allen et al., 2011; Hayter and Brown, 2018)] and albino rats [SCN: (Meijer et al., 1998; Drouyer et al., 2007; Tsuji et al., 2016); OPN: (Szkudlarek et al., 2012; Orlowska-Feuer et al., 2016)]. For the black hooded rat there is one published study investigating the OPN (Trejo and Cicerone, 1984) and one for the SCN (Aggelopoulos and Meissl, 2000). In contrast, rich literature exists covering light responsive cells in the dLGN of different species [mouse: (Brown et al., 2010, 2012; Piscopo et al., 2013; Denman et al., 2017; Stabio et al., 2018; Román Rosón et al., 2019); black hooded rats: (Sriram et al., 2016; Jeczmien-Lazur et al., 2019)].

For the classification of light-responsive cells an ambiguous and varied nomenclature is used within the literature, varying by brain region and response complexity. Generally, light responsive cells are categorized as transient and tonic and sometimes further depending on whether they show an increase (ON) or decrease (OFF) in spiking upon light presentation. Additionally some authors also distinguish “delayed cells” as a separate category, however, these responses are likely not monosynaptically driven by the retina. Because we aimed to classify light responsive cells in all three structures in the same way, we have adopted a mixed nomenclature between that used for the dLGN (Brown et al., 2010, 2012; Román Rosón et al., 2019), SCN (Groos and Mason, 1980; Brown et al., 2011) and the retina (Krieger et al., 2017). We categorized cells as transient and tonic and further depending on whether they responded to light increment or decrement as transient ON and OFF and sustained and suppressed. Thus, our classification resulted in four categories of cells, examples of each were recorded in all brain structures investigated.

Interestingly, however, there were some differences in the proportion of different types of cells between structures, most probably reflecting different retinal input and/or structure function (Brown et al., 2010; Schmidt et al., 2011; Do, 2019; Román Rosón et al., 2019). In the case of the dLGN and OPN, our results are in agreement with previously published data showing the majority of recorded cells as transient (dLGN) and sustained (OPN) (Trejo and Cicerone, 1984; Brown et al., 2010, 2011; Allen et al., 2011; Szkudlarek et al., 2012; Jeczmien-Lazur et al., 2019; Román Rosón et al., 2019). However, our data reveal some differences in the SCN, as we have found mostly transient neurons. In previous SCN recordings in albino (Groos and Mason, 1980; Meijer et al., 1998; Tsuji et al., 2016) and pigmented (Aggelopoulos and Meissl, 2000) rats and mice (Brown et al., 2011; van Diepen et al., 2013) the majority of recorded light responsive cells were sustained. Sustained and suppressed characteristics in the SCN matches the anatomy, as it is known that retinal input to the mice SCN is in majority (if not only) build up by melanopsin cells (Baver et al., 2008; Brown et al., 2010; Ecker et al., 2010; Chen et al., 2011; Schmidt and Kofuji, 2011). It is possible, however, that some species differences exist, and in black hooded *Long Evans* rats the retino-hypothalamic tract has a different structure to that in mice, or that electrophysiological variations are present. In fact, it has already been shown that rat M1 cells [one of the first two types of melanopsin cells described and known to predominantly signal light to the biological clock (Berson et al., 2002; Baver et al., 2008; Ecker et al., 2010)] have far more transient responses than mice (Reifler et al., 2015). It has also been suggested that this electrophysiological discrepancy may result in the different photoentrainment thresholds observed in mice and rats (Reifler et al., 2015). Our results are in line with these observations and suggest that light responses in the pigmented rat SCN are more transient than in mice [however, it is important to note the differences in light intensities used between the present study (log 13.7 photons/cm^2^/s) and other published work (log 15 photons/cm^2^/s or higher) (Meijer et al., 1989; Aggelopoulos and Meissl, 2000; Brown et al., 2011)]. On the other hand, such differences may also result from anatomical localization of the recorded cells, as in the current study cells were mostly positioned in the rostral SCN near the third ventricle (Figure 2). Additionally, it is important to highlight the limitations of the current study in limited n numbers, although each cell type was represented in our sample.

### Removing Short Wavelengths From Polychromatic Light Attenuates Light-Induced Neuronal Activity in Different Types of Neurons in the dLGN and OPN

The main aim of this work was to verify whether light induced neuronal activity in retinorecipient brain structures is affected by removing short wavelengths (UV-blue) from the polychromatic white light spectrum. The amber lens filters 90% of short wavelengths (cut off at 525 nm), thus mostly reduces activity of S-cones and melanopsin, having maximal sensitivity at approximately 359 and 480 nm, respectively, (Jacobs et al., 2001, 2003; Lucas et al., 2014). Intuitively one would expect that such UV/blue light blockade should have profound consequences on the light induced activity. In fact, we observed attenuation of neuronal activity in transient ON and sustained cells in the dLGN and OPN, respectively, We have made an attempt to verify that also in the SCN, however, small numbers of cells were recorded. Nonetheless, in both the dLGN and SCN, were cells which stopped responding to filtered light suggesting exclusive S-cone input. It is generally thought that transient cells receive retinal input from rods and cones that drive fast onset/offset peak activity. On the other hand, sustained and suppressed cells receive input from all three classes of photoreceptors, because ipRGCs act as conduit for signals originating from classic photoreceptors. Also, it was recently revealed that melanopsin cells can be GABAergic (Sonoda et al., 2020). In this way different phases of the light responses are identified with signals from different photoreceptor classes (Mure et al., 2007; Wong et al., 2007; Brown et al., 2010; Allen et al., 2011). The current results show that in the dLGN only neurons receiving signal from classic photoreceptors are influenced, thus most probably those which are engaged in some aspects of vision formation (e.g., contrast, color discrimination, visual acuity, motion etc.). In contrast, in the OPN decreased activity was observed in sustained cells, known to code ambient irradiance (Allen et al., 2011; Brown et al., 2011, 2012; Wong, 2012). The OPN is best known for pupil control (Trejo and Cicerone, 1984), however, there are strong suggestions that it also contributes to circadian rhythm regulations. It plays a role in triggering of rapid eye movement sleep in response to shift from light to darkness (Miller et al., 1998) and is responsible for masking effects (Gall et al., 2017). Moreover, color opponent neurons in the OPN were suggested to contribute to other NIF functions than pupillary control (Hayter and Brown, 2018). These results match anatomical data showing that retinal input to each of the investigated structures is comprised of not only different types of retinal ganglion cells [∼thirty types known (Sanes and Masland, 2015; Román Rosón et al., 2019)], but also of melanopsin cells [six subtypes of ipRGCs described, termed M1-6 (Schmidt et al., 2011; Do, 2019)]. The retinal input to the SCN primarily derives from melanopsin cells with the majority Brn3b-negative M1, whereas the OPN receives input from Brn3b-positive M1 and M2 cells (Chen et al., 2011). In contrast, the dLGN is mostly innervated by non-melanopsin retinal ganglion cells (Sanes and Masland, 2015) and non-M1 melanopsin cells with significant contribution of M4, M5, and M6 (Estevez et al., 2012; Stabio et al., 2018; Quattrochi et al., 2019). Thus, observed differences in the types of neurons affected by short wavelength removal may be due to variations in subtype composition projecting to these regions, reflecting the physiological diversity of melanopsin cells.

It is important to highlight that even though each photoreceptor class has spectrally distinct peaks, their spectral sensitivities extensively overlap (Figure 1B; Spitschan and Woelders, 2018). Moreover, according to the *principle of univariance* (Rushton, 1972), photoreceptors cannot distinguish between changes in irradiance and changes in wavelength. As a consequence, most light sources, even monochromatic light, as used in this study (UV), activate all photoreceptors to some degree (Figure 1C). Thus, removing short wavelengths from polychromatic light does not mean that S-cones and melanopsin are not activated (Figure 1C) but rather that they are activated to a lesser degree. Moreover, such change in spectrum results in a change in overall irradiance. In fact, in this study S-cone activation was reduced by 2 logs and melanopsin by 1 log between white and filtered light. Moreover, due to photoreceptor input differences there may be consequential spectral and irradiance sensitivity differences between investigated structures. The present results show no changes in the peak activity of transient ON cells in the OPN, but this group of cells is affected the most in the dLGN. It may reflect weaker S-cone input to NIF centers in comparison to image forming visual structures in black hooded rats. Similar findings were previously reported in mice (Brown et al., 2011). Importantly, when the irradiance was matched, we did observe differences in the amplitude of peak activity between white and filtered light (Figure 7).

Such light filtration not only changes light spectrum and irradiance but also strongly impacts perceived color (changing from white to orange). Recent studies have shown that color-opponent neurons are widely found in the mouse dLGN (Denman et al., 2017), SCN (Walmsley et al., 2015) and OPN (Hayter and Brown, 2018) and that mice use color as a cue for photoentrainment (Walmsley et al., 2015). Although a similar study identifying color opponent neurons in these regions has yet to be conducted in pigmented rats it is possible that observed responses in the dLGN and OPN in the present study may be due to variations in color. Several human studies have investigated different visual attributes (visual acuity, contrast sensitivity, depth perception, low-contrast visual acuity, dynamic visual acuity, and hand-eye coordination) when wearing tinted lenses. These have revealed contradictory findings (Kuyk and Thomas, 1990; Wolffsohn et al., 2000; Pérez et al., 2003; Porisch, 2007; Kohmura et al., 2013), however, changes in color discrimination seems to be a common finding (Kuyk and Thomas, 1990; De Fez et al., 2002; Kohmura et al., 2013).

Differences in the neuronal response profile were observed between white and filtered light. Three main types of neurons were found: stable, changing response profile and not responding to filtered white light. Interestingly, all three types were found in the dLGN, stable and not responding in the SCN and stable and changing the response profile in the OPN. Differences in the response profile may reflect changes in the irradiance. In fact, the dLGN has previously been attributed such a feature (Tikidji-Hamburyan et al., 2014) and this could potentially exist in the OPN. Another plausible explanation is that neurons which stopped responding after removing short wavelengths may receive only S-cone input. These retinal ganglion cells have previously been shown to exist (Ekesten et al., 2000; Ekesten and Gouras, 2005). There are at least three types of dLGN cells receiving different retinal inputs in mice. Among them are so called relay-mode cells integrating information from up to five retinal ganglion cells of the same type (Rompani et al., 2017). We speculate that dLGN cells not responding to filtered light are potentially relay-mode cells receiving input from S-cones through a single type of RGC. Existence of such cells in the SCN would be surprising, and definitely requires further studies, however, again we cannot rule out the possibility that species differences exist.

Assuming that sustained OPN cells changing their response profile from sustained to transient in response to filtered light would have stronger S-cone than melanopsin input, we hypothesized that some differences in irradiance coding properties exist between them. Interestingly, both groups were fitted with the same response curve suggesting that, at least in the irradiance range tested here, there are no differences between these cell types. It has previously been revealed that S-cones support sustained activation in the mouse OPN (Allen et al., 2011) and SCN (Oosterhout et al., 2012; van Diepen et al., 2013) and that UV light modulates circadian behavior and sleep in melanopsin knockout mice (Van Oosterhout et al., 2012).

### Neurons in the Rat dLGN and SCN Are Sensitive to UV Light

Murine retinae have two types of cone opsin (SVS and MVS) and their lens only provides a modest barrier to UV absorption [Figure 2 (Jacobs et al., 1991; Szél and Röhlich, 1992; Szel et al., 1993)]. Taking all this into account it appears that rats have the capacity to see UV light and utilize it for color vision. There is some evidence that rats are able to perform color discrimination tasks (Jacobs et al., 2001) and electroretinogram measurements have shown prominent S-cone signals under chromatic adaptation (Jacobs et al., 1991) with similar results also observed in the SCN (Aggelopoulos and Meissl, 2000). Results of the present study go some way in bridging the gap in electrophysiological data by showing that neurons in the black hooded rat dLGN and SCN are responsive to monochromatic UV light stimulation as dim as 9.95 log S-cone photons/cm^2^/s. This is consistent with previous electrophysiological reports (Lall et al., 2010; Allen et al., 2011), but significantly lower than described for S-cones pupil responses in mice (Yao et al., 2006). Interestingly, UV light stimulation evoked the same four types of light responses as polychromatic white light, in both the dLGN and SCN. Even though monochromatic light (360 nm) with a very narrow peak was used (Figure 1A), it has to be acknowledged that this would still excite other photoreceptors (Figures 1B,C). Melanopsin contribution, however, can be ruled out as its activation threshold [11 – 12 log photons/cm^2^/s (Lall et al., 2010)] is above that used in the current study. Therefore, the observed responses are likely elicited by rods and cones in the dLGN and cones in SCN, as rods were showed to have negligible effect in that structure. In favor of cones are observations of sustained photoresponses, which are only elicited by S-cones and melanopsin (Mure et al., 2007; Wong et al., 2007; Brown et al., 2010; Allen et al., 2011). Here we did not focus on the spectral sensitivity of retinorecipient structures, nevertheless this subject requires further investigation and would be an interesting addition to the current study. In mice there exists a plethora of data on S-cone input to various retinorecipient structures. Definitely mice not only possess color opponent neurons in different parts of the visual system (Walmsley et al., 2015; Denman et al., 2017; Hayter and Brown, 2018), but also use UV light (Oosterhout et al., 2012; van Diepen et al., 2013) and spectral changes (Walmsley et al., 2015) to synchronize their activity to the light dark cycle and discriminate colors (Denman et al., 2018).

## Conclusion

We found that removing short-wavelengths from polychromatic light attenuates the most transient ON and sustained cells in the dLGN and OPN, respectively. We link the type of cells with their possible function and thus speculate that such change in the spectrum influences both image (transient ON cells) and non-image (sustained cells) forming functions. Moreover, we provide electrophysiological evidence that rats are sensitive to UV light by showing neuronal responses to even very dim monochromatic light (360 nm) in the dLGN and SCN. Finally, we compare the ability of different types of sustained OPN neurons, either adapting or maintaining their response profile in response to filtered polychromatic light to irradiance coding, and show that both groups do it equally effective and thus, we conclude that S-cones may provide information about ambient irradiance.

The current results confirm that neuronal activity in retinorecipient structures is affected by removing short wavelengths from polychromatic light. Thus, it strongly suggests that this forms the basis of observed differences in both visual and circadian functions in behavioral animal (and human) studies.

## Data Availability Statement

The raw data supporting the conclusions of this article will be made available by the authors, without undue reservation.

## Ethics Statement

The animal study was reviewed and approved by the Ethics Committee of Jagiellonian University in Krakow (permission no.: 25/2013).

## Author Contributions

PO-F and MHL conceived the project. PO-F designed the experiments. PO-F, MKS, and AA performed the experiments. PO-F analyzed and interpreted the data and wrote the manuscript. All authors revised the manuscript critically and approved the final version.

## Conflict of Interest

The authors declare that the research was conducted in the absence of any commercial or financial relationships that could be construed as a potential conflict of interest.
